# Identification and Characterization of Pathogens Causing Sugarcane (*Saccharum officinarum* L.) Leaf Spot and Screening for Antagonistic Bacteria

**DOI:** 10.3390/jof12060384

**Published:** 2026-05-26

**Authors:** Lianghui Jiang, Kunfa Gan, Jinlan Xie, Zhanghong Mo, Qiang Liang, Xing Huang, Qian Nong, Li Lin, Changning Li

**Affiliations:** 1 Key Laboratory of Sugarcane Biotechnology and Genetic Improvement (Guangxi), Ministry of Agriculture and Rural Affairs/Guangxi Key Laboratory of Sugarcane Genetic Improvement, Nanning 530007, China; 18788652073@163.com (L.J.); gankunfa0922@163.com (K.G.); xiejinlan2008@126.com (J.X.); mozhanghong@126.com (Z.M.); liangqiangde@163.com (Q.L.);; 2 Guangxi Key Laboratory of Biology for Crop Diseases and Insect Pest, Plant Protection Research Institute, Guangxi Academy of Agricultural Sciences, Nanning 530007, China

**Keywords:** sugarcane, leaf spot, *Epicoccum latusicollum*, *Fusarium sacchari*, biological characteristics, antagonistic bacteria

## Abstract

Sugarcane is a globally important crop, widely cultivated for sugar production and bioenergy. However, leaf spot disease leads to a reduction in its quality and yield. In this study, pathogen identification, biological characteristic analysis, and screening of antagonistic bacteria against the causal pathogens were done as a basis for epidemic prediction and green control of sugarcane leaf spot disease. The causal pathogens of sugarcane leaf spot disease were identified as *Epicoccum latusicollum* El532 and *Fusarium sacchari* Fs64, respectively, based on morphological characteristics, multi-gene phylogenetic analysis (*ITS*, *TUB2*, and *RPB2* for El532; *ITS*, *TEF1α*, and *RPB2* for Fs64), and pathogenicity tests. Biological characterization revealed that both pathogens exhibited optimal mycelial growth at 25 °C and under continuous darkness. However, light-dark cycles inhibited their growth. The optimal pH ranges for both isolates were 6–9 and 5–10, respectively. Maltose was the optimal carbon source for El532, whereas maltose, lactose, and starch were optimal for Fs64. Yeast extract served as the optimal nitrogen source for both. Isolation and screening of bacterial strains from healthy sugarcane roots, leaves, and rhizosphere soil yielded 13 antagonistic bacterial strains. Among them, six strains exhibited inhibition rates exceeding 57% against both pathogens. *Bacillus subtilis* A5 exhibited the highest antagonistic activity (68.85% against El532, 71.69% against Fs64), underscoring its potential as a promising biocontrol candidate. These findings provide a scientific basis for the diagnosis and management of sugarcane leaf spot disease.

## 1. Introduction

Sugarcane (*Saccharum officinarum* L.) is one of the most important economic crops worldwide. It primarily serves as a source of sugar and is also a key raw material for bioethanol production. Notably, its cultivation efficiency is directly linked to the development of the sugar industry and advancement of biomass energy production [1,2]. Sugarcane is mainly cultivated in tropical and subtropical regions, with China being one of the major producing countries, after India and Brazil [3]. However, its production is often threatened by various fungal diseases, including pokkah boeng, smut, red rot, and leaf spot, during its growth cycle [4,5]. Notably, leaf spot, which is typically characterized by necrotic lesions on leaves, is a prevalent foliar disease occurring in major sugarcane-producing regions, including China (e.g., Guangxi, Guangdong and Fujian), India, and Brazil. It directly impairs photosynthesis, which is a critical physiological process for sugar accumulation in sugarcane plants [6], consequently severely restricting the quality and yield of sugarcane [4,7], with yield losses up to 50% [8]. The incidence of sugarcane leaf spot has increased in recent years because of the continuous expansion of the planting scale and popularization of continuous cropping systems, thereby posing a severe challenge to the sustainable development of the sugarcane industry.

Sugarcane leaf spot is caused by various phytopathogenic fungi. Studies have shown that sugarcane foliar diseases are caused by various fungal genera worldwide, including *Fusarium*, *Epicoccum*, *Curvularia*, *Puccinia*, and *Phoma* [7,8,9,10,11]. *Fusarium* species are important pathogens causing pokkah boeng and other diseases, while *Epicoccum* species are a major group causing sugarcane leaf spot. Notably, both *Epicoccum* and *Fusarium* species are common pathogens causing leaf spot diseases in various crops, such as walnut (*Juglans regia* L.), sweet cherry (*Prunus avium* L.), maize (*Zea mays* L.), and Italian ryegrass (*Lolium multiflorum* Lam.) [12,13,14,15]. Previous studies have conducted identification and pathogenicity analyses of certain fungal pathogens associated with sugarcane leaf spot. However, there are no systematic investigations on the pathogen species causing sugarcane leaf spot disease in Guangxi, which is the largest sugarcane-producing region in China, accounting for 0.77 Mha of sugarcane area and 41.22 MT of millable cane yield in the 2022/2023 milling season [16]. Accurate identification of pathogens is crucial for formulating effective disease management strategies. Moreover, understanding the biological characteristics of pathogens, including their responses to environmental factors, such as temperature, pH, carbon and nitrogen sources, and light conditions, is crucial for predicting disease occurrence patterns and optimizing control measures [17]. However, there are limited studies on the biological characteristics of pathogens associated with sugarcane leaf spot.

Chemical fungicides, such as fluxapyroxad, pyraclostrobin, and metconazole, have long been the primary measure for the control of sugarcane leaf spot [8]. However, their extensive application has led to several critical issues such as pathogen resistance, ecological environmental damage, and pesticide residues [18,19]. Biocontrol using antagonistic microorganisms has emerged as an important alternative to chemical control because it is safer, efficient, and environmentally sustainable [20]. In particular, antagonistic bacteria are the most promising biocontrol resources because of their rapid reproduction, strong environmental adaptability, and diverse antagonistic mechanisms, including antibiotic production, nutrient competition, and induction of host resistance [21]. Antagonistic bacteria against sugarcane smut, red rot, and *Fusarium* wilt have been screened and documented. For instance, *Bacillus velezensis*, *Pseudomonas guariconensis*, and *Burkholderia gladioli* are effective in controlling sugarcane smut caused by *Sporisorium scitamineum* [22,23,24]. *Pseudomonas putida* and *B. velezensis* exhibit biocontrol effects on sugarcane red rot caused by *Glomerella tucumensis* and *Colletotrichum falcatum*, respectively [25,26]. *Bacillus inaquosorum* and *Bacillus vallismortis* are proposed as potential biocontrol agents for sugarcane *Fusarium* wilt caused by *Fusarium sacchari* [27]. However, screening of antagonistic bacteria against sugarcane leaf spot pathogens, especially *Epicoccum* and *Fusarium* species, remains largely unexplored. Moreover, there are limited reports on the biocontrol of sugarcane leaf spot.

In this study, sugarcane leaves showing typical leaf spot symptoms were subjected to pathogen isolation and purification. Precise species identification of the pathogens was subsequently performed based on morphological characteristics and multi-gene sequence analysis. Their environmental adaptability was assessed by systematically evaluating the effect of temperature, pH, light, carbon sources, and nitrogen sources on mycelial growth. In addition, antagonistic bacteria were isolated and screened from healthy sugarcane tissues and rhizosphere soil. This study aimed to identify the causal pathogens of sugarcane leaf spot in Guangxi, China, their biological characteristics, and screen potential biocontrol bacterial resources. The findings of this study provide a theoretical basis and technical support for accurate identification, epidemic prediction, and green control of sugarcane leaf spot.

## 2. Materials and Methods

### 2.1. Occurrence of Leaf Spot in Sugarcane and Sample Collection

**Figure 1:**
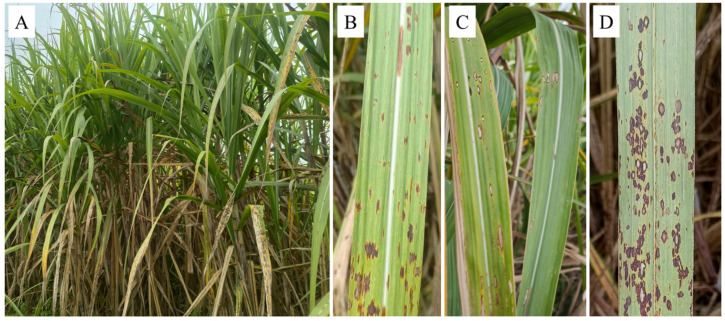
Occurrence and symptoms of sugarcane leaf spot in the field. (**A**) Occurrence of leaf spot in the field. (**B**–**D**) Typical symptoms of leaf spot.

Sugarcane plants exhibiting typical symptoms of leaf spot disease were observed in the experimental field of the Sugarcane Research Institute, Guangxi Academy of Agricultural Sciences, Nanning, Guangxi (22°50′ N, 108°14′ E) in October 2025. The disease occurred in a patchy to aggregated distribution pattern, with an incidence of over 50%. Small pale yellow or chlorotic spots initially appeared on sugarcane leaves, but gradually expanded into irregular or subcircular lesions of varying sizes as the disease progressed. The lesion center turned tawny, brown, and dark brown, with distinct yellowish-green halos at the margins, exhibiting a striking contrast with healthy leaf tissues. Individual lesions coalesced to form large necrotic patches during the severe disease phase, leading to scorching and yellowing of local leaf tissues and premature senescence of entire leaves (Figure 1).

Diseased leaf samples with typical leaf spot symptoms were randomly collected from different sugarcane clumps in the experimental field. The samples were placed into sterile, sealed plastic bags and transported to the laboratory, which was used for pathogen isolation and identification. Asymptomatic sugarcane leaves, roots, and rhizosphere soil were also collected for isolation and screening of antagonistic bacteria. All samples were processed within 24 h of collection to ensure optimal isolation efficiency.

### 2.2. Pathogen Isolation and Purification

Small tissue pieces (approximately 5 × 5 mm) were excised from the margins between symptomatic and healthy leaf sections using a sterile scalpel. The pieces were sequentially surface-sterilized with 75% ethanol (30 s) and 2% NaClO (2 min), then rinsed five times with sterile distilled water. The disinfected pieces were placed on potato dextrose agar (PDA) plates (containing 200 g/L potato infusion, 20 g/L glucose, and 20 g/L agar) supplemented with 50 mg/L cefotaxime sodium and incubated in a dark 28 °C incubator (Yiheng Scientific Instrument, Shanghai, China). Hyphal tips from colony margins were subcultured on fresh PDA plates at least three times to obtain pure cultures, which were maintained in a 4 °C refrigerator. Isolates El532 and Fs64 were selected for further analysis.

### 2.3. Pathogenicity Testing

The pathogenicity of fungal isolates El532 and Fs64 was evaluated using a pot experiment. Healthy and asymptomatic potted sugarcane plants (six-month-old) were selected, wounded with a sterile needle, and then inoculated by spraying with 3 mL of conidial suspension (1 × 10^7^ conidia/mL) using a spray bottle. The control plants were sprayed with an equal volume of sterile water. Symptom development was observed daily for 7 d. Re-isolation was performed from typical lesions at 7 d post-inoculation. The recovered fungi were morphologically compared with the original isolates, El532 and Fs64, to fulfill Koch’s postulates.

### 2.4. Pathogen Identification

#### 2.4.1. Morphological Identification

Isolates El532 and Fs64 were inoculated onto PDA plates and incubated in a 28 °C dark incubator. Colonial morphological characteristics were observed at 5 and 15 days of incubation. Small amounts of mycelia and conidia were subsequently mounted in sterile water on glass slides for microscopic observation. The morphological characteristics of the mycelia and conidia were examined under a light microscope (Nikon Corporation, Tokyo, Japan). Conidial dimensions were measured, with at least 50 conidia measured per isolate.

#### 2.4.2. Molecular Identification

Genomic DNA was extracted from fresh mycelia of isolates El532 and Fs64 using an Ezup column fungi genomic DNA purification kit (Sangon Biotech, Shanghai, China) following the manufacturer’s instructions and stored in a −20 °C refrigerator. The *internal transcribed spacer* (*ITS*) region, *RNA polymerase II second largest subunit* (*RPB2*), and *β-tubulin* (*TUB2*) genes of isolate El532 were amplified. In contrast, *ITS*, *RPB2*, and *translation elongation factor 1-α* (*TEF1α*) genes of isolate Fs64 were amplified. All primers used were synthesized by Sangon Biotech and are listed in Table 1. PCR amplification was performed in a 50 μL reaction containing 25 μL of 2 × GS Taq PCR Mix, 2 μL each of forward and reverse primers, 2 μL of genomic DNA template, and 19 μL of double-distilled water. The PCR program was set as follows: initial denaturation at 95 °C for 3 min; 35 cycles of denaturation, annealing, and extension at 94 °C for 25 s, the optimal temperature for 25 s, and 72 °C for 10 s, respectively; and a final extension at 72 °C for 5 min. The PCR products were subsequently sent to Sangon Biotech for sequencing. The obtained sequences were subjected to Basic Local Alignment Search Tool (BLAST) analysis in the National Center for Biotechnology Information (NCBI) database. Reference sequences of the closely related species were downloaded from GenBank. Concatenation of the multi-gene sequences of El532 and Fs64 was done separately, followed by multiple sequence alignment using MEGA 7.0 software. A phylogenetic tree was constructed using the maximum likelihood (ML) method with 1000 bootstrap replicates.

### 2.5. Biological Characteristics of Isolates El532 and Fs64

#### 2.5.1. Carbon and Nitrogen Sources

The effects of different carbon and nitrogen sources on mycelial growth of isolates El532 and Fs64 were evaluated using Czapek’s medium (3 g/L NaNO_3_, 1 g/L KH_2_PO_4_, 0.5 g/L MgSO_4_·7H_2_O, 0.5 g/L KCl, 0.01 g/L FeSO_4_·7H_2_O, 20 g/L sucrose, 20 g/L agar) as the basal medium. Carbon source utilization assay was performed by replacing sucrose in the basal medium with various carbon sources (including glucose, starch, sodium carboxymethyl cellulose (CMC-Na), lactose, mannitol, maltose, sorbitol, xylose, and fructose), while nitrogen source utilization assay was performed by replacing NaNO_3_ with various nitrogen sources (including peptone, yeast extract, urea, (NH_4_)_2_SO_4_, glycine, beef extract, tryptophan, casein, and glutamic acid). Media without carbon or nitrogen sources served as blank controls. Mycelial plugs (7 mm diameter) of El532 and Fs64 colonies were placed at the center of each plate and incubated in a 28 °C dark incubator. The colony diameter was measured using the cross method after 5 days. Each treatment was replicated three times.

#### 2.5.2. Temperature and pH

The effects of temperature and pH on the mycelial growth of isolates El532 and Fs64 were evaluated using Czapek’s medium. Mycelial plugs were cut from the margins of actively growing colonies and placed at the center of Czapek’s agar plates. For temperature assays, plates were incubated at 5–35 °C (5, 10, 15, 20, 25, 28, 30, and 35 °C) in the dark. For pH assays, the Czapek’s medium pH was adjusted to 4.0–11.0 (4.0, 5.0, 6.0, 7.0, 8.0, 9.0, 10.0, and 11.0) using 1 mol/L HCl or NaOH prior to sterilization. Mycelial plugs were then inoculated and incubated in a 28 °C dark incubator. The colony diameter was measured using the cross method after 5 days. Each treatment was replicated three times.

#### 2.5.3. Light Regime

The effects of different light regimes on the mycelial growth of isolates El532 and Fs64 were evaluated on Czapek’s medium. Mycelial plugs were inoculated at the center of the plates and incubated in a 28 °C incubator under continuous light (24 h light), continuous darkness (24 h dark), and alternating light/dark (12 h light/12 h dark). The colony diameter was measured using the cross method after 5 days. Each treatment was replicated three times.

### 2.6. Isolation and Screening of Antagonistic Bacteria

Isolation and purification of antagonistic bacteria was done on LB medium (10 g/L tryptone, 5 g/L yeast extract, 10 g/L NaCl, 20 g/L agar). Leaf and root tissue samples were surface sterilized as described in Section 2.3, then ground in sterile saline solution (0.85% NaCl). Rhizosphere soil samples (10 g) were mixed with 90 mL of sterile saline solution and shaken on a shaker at 180 rpm for 30 min at room temperature. The resulting tissue homogenate and soil suspension were serially diluted (10-fold) with sterile saline solution. Aliquots (50 μL) of each dilution were spread on LB agar plates, after which the plates were incubated in a 28 °C dark incubator for 24 h. Bacterial colonies with different morphological characteristics were selected and purified by repeated streaking on fresh LB plates. Pure cultures were preserved in glycerol at −80 °C in an ultra-low temperature freezer (Thermo Scientific, Waltham, MA, USA).

The dual culture method was employed to screen bacteria with antagonistic activity against El532 and Fs64. Bacterial strains were cultured in LB broth in an incubator-shaker (Fosfu, Hebei, China) set at 28 °C and 180 rpm until the logarithmic growth phase. A mycelial plug of each pathogen was placed at the center of a PDA plate, and the bacterial culture was streaked on both sides approximately 2 cm away from the center. Plates inoculated with the pathogen only served as controls. Each treatment had three biological replicates. All plates were incubated in a 28 °C dark incubator for 5 days, after which the colony diameter of the pathogen was measured. The inhibition rate was calculated using the following formula: Inhibition rate (%) = [(D_1_ − D_2_)/D_1_] × 100, where D_1_ represents the colony diameter of the pathogen in the control plate, and D_2_ represents the colony diameter of the pathogen toward the bacterial streak in the treatment plate.

### 2.7. Identification of Antagonistic Bacteria

Bacterial strains exhibiting significant antagonistic activity were selected for identification based on 16S rRNA gene sequencing. Genomic DNA was extracted using a bacterial genomic DNA extraction kit (Solarbio, Beijing, China) following the manufacturer’s instructions and stored at −20 °C awaiting use. The *16S rRNA* gene was amplified using the universal primer pair 27F and 1492R, synthesized by Sangon Biotech. PCR amplification was performed in a 50 μL reaction system containing 25 μL of 2 × GS Taq PCR Mix, 2 μL each of forward and reverse primers, 2 μL of DNA template, and 19 μL of ddH_2_O. The PCR program was set as follows: initial denaturation at 95 °C for 3 min; 35 cycles of denaturation, annealing, and extension at 94 °C for 25 s, 55 °C for 25 s, and 72 °C for 10 s, respectively; and a final extension at 72 °C for 5 min. The PCR products were verified by 1% agarose gel electrophoresis and sequenced by Sangon Biotech. The obtained sequences were submitted to the NCBI database and analyzed using BLAST to identify closely related bacterial species.

### 2.8. Statistical Analysis

Data are expressed as means ± standard deviation (SD) of three biological replicates. Data collation and preliminary calculations were conducted using Microsoft Excel 2013. Statistical analysis was performed using IBM SPSS Statistics 27.0. One-way analysis of variance (ANOVA) followed by the least significant difference (LSD) test was used to determine the significant differences among treatments at a threshold of *p* < 0.05. All figures were generated using GraphPad Prism 8.0.1 and Microsoft PowerPoint 2013.

## 3. Results

### 3.1. Pathogen Isolation and Pathogenicity Testing on Sugarcane

**Figure 2:**
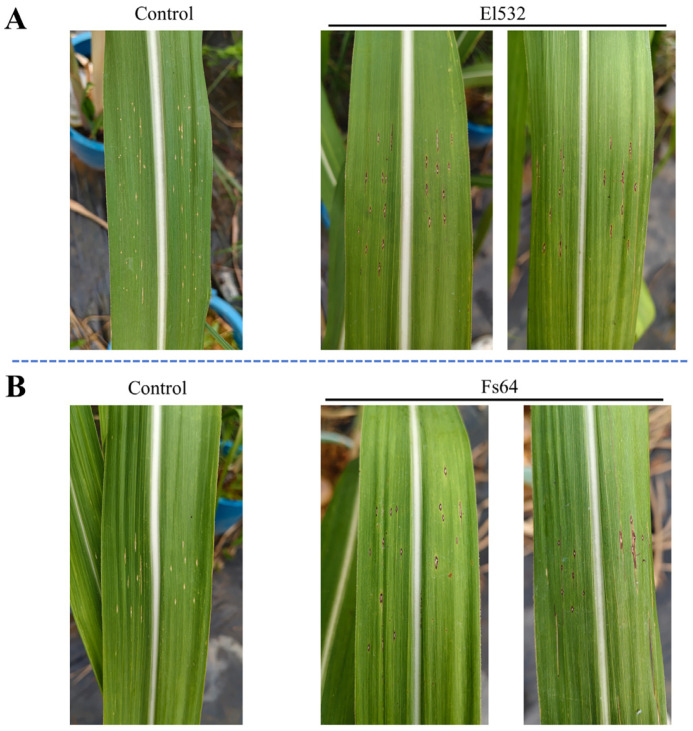
Pathogenicity analysis of isolates El532 and Fs64 on sugarcane leaves. (**A**) Pathogenicity analysis of isolate El532. (**B**) Pathogenicity analysis of isolate Fs64. The control represents leaves inoculated with sterile water, while El532 and Fs64 represent the inoculated leaves.

A total of 11 fungal isolates were obtained from diseased sugarcane leaf samples and were primarily classified into two morphotypes, designated as El532 and Fs64, based on preliminary morphological characteristics. Pathogenicity testing of the two isolates revealed that sugarcane leaves inoculated with either of the isolates developed typical leaf spot symptoms, consistent with those observed in the field (Figure 2). Small chlorotic spots and irregular brown necrotic lesions appeared on the leaf surface, and the lesion areas gradually expanded with incubation time. Control leaves remained asymptomatic throughout the experimental period. Fungi re-isolated from the lesions of inoculated leaves exhibited colonial and microscopic characteristics consistent with the original inoculated isolates El532 and Fs64, thereby fulfilling Koch’s postulates. These findings confirmed that El532 and Fs64 are the causal pathogens of sugarcane leaf spot and exhibit strong pathogenicity to sugarcane.

### 3.2. Colony and Microscopic Morphology of the Pathogens

**Figure 3:**
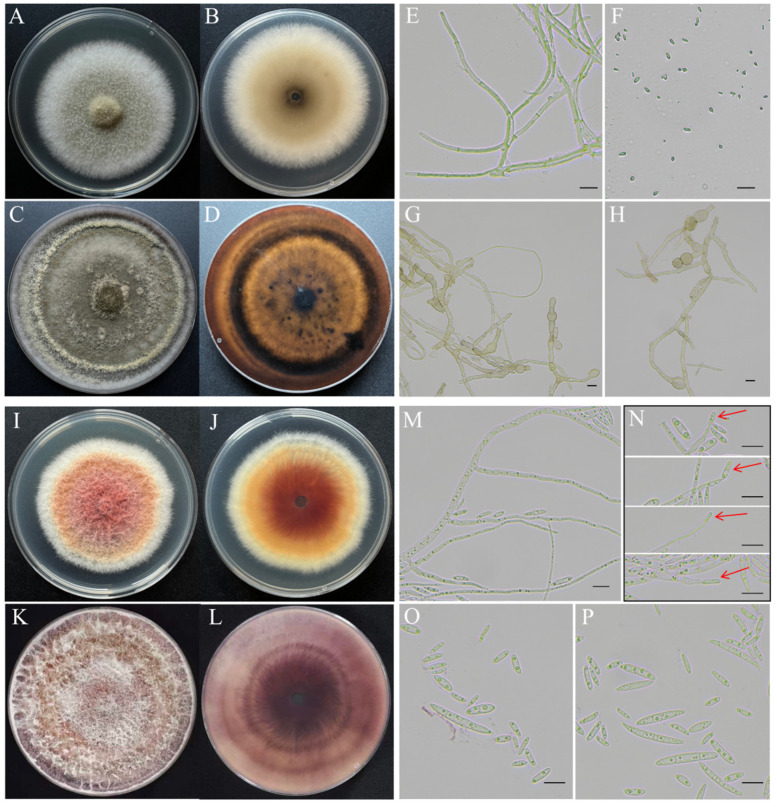
Colony morphology on PDA medium and microscopic morphology of hyphae and conidia of isolates El532 and Fs64. (**A**,**B**) Front and reverse sides of El532 cultured on PDA for 5 days. (**C,D**) Front and reverse sides of El532 cultured on PDA for 15 days. (**E**) Hyphae of El532. (**F**) Conidia of El532. (**G**,**H**) Chlamydospores of El532. (**I**,**J**) Front and reverse sides of Fs64 cultured on PDA for 5 days. (**K**,**L**) Front and reverse sides of Fs64 cultured on PDA for 15 days. (**M**) Hyphae of Fs64. (**N**) Microconidia forming false heads of Fs64. (**O**,**P**) Macroconidia and microconidia of Fs64. The red arrow indicates microconidia forming false heads of Fs64. Scale bars = 10 μm.

Colonies of isolate El532 exhibited a cottony appearance with abundant aerial mycelia after 5 days of incubation on PDA. The colonies were initially white to gray or light brown, with a brown to light red reverse (Figure 3A,B). However, the colonies turned entirely gray or light brown, with reddish-orange pigment deposition on the reverse side after 15 days (Figure 3C,D). Microscopic observation revealed hyaline, branched, septate hyphae (Figure 3E). Conidia were ellipsoidal or ovoid, aseptate, hyaline, measuring 3.7–6.1 × 1.5–2.9 μm (*n* = 50) (Figure 3F). Chlamydospores were brown, subglobose, unicellular or multicellular, measuring 11.2–24.8 × 9.7–22.6 μm (*n* = 50) (Figure 3G,H). In contrast, colonies of isolate Fs64 exhibited fluffy aerial hyphae, appearing pink, orange, and white from the center to the margin, with a reddish-brown center on the reverse side after 5 days of incubation on PDA (Figure 3I,J). However, the hyphae became dense, with a white to orange surface, while the reverse showed dark purple or purplish-red pigments after 15 days (Figure 3K,L). Microscopically, the hyphae were hyaline, branched, and septate (Figure 3M). Two types of conidia were observed, the macroconidia were slender, slightly falcate, with 2–5 septa, measuring 20.1–46.3 × 2.4–5.1 μm (*n* = 50). In contrast, the microconidia were ellipsoidal or reniform, 0–1 septate, measuring 5.2–11.3 × 1.9–3.8 μm (*n* = 50), forming false heads on monophialides (Figure 3N–P). Isolates El532 and Fs64 were thus preliminarily identified as *Epicoccum* sp. and *Fusarium* sp., respectively, based on these morphological characteristics.

### 3.3. Molecular Identification and Phylogenetic Analysis

**Figure 4:**
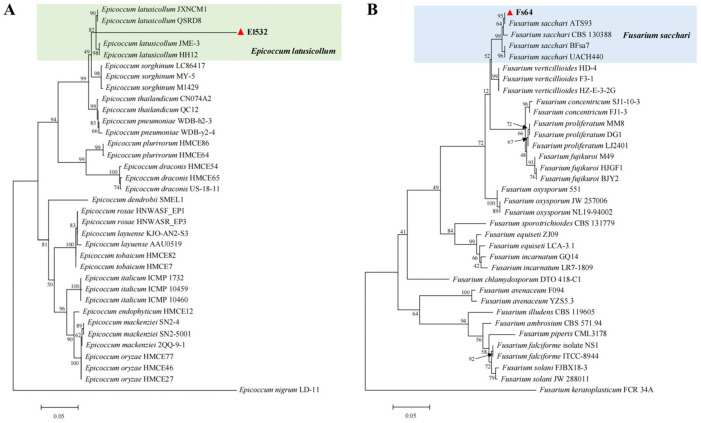
Phylogenetic trees of El532 and Fs64 constructed using the maximum likelihood method based on concatenated multi-gene sequences. (**A**) Phylogenetic tree of El532 based on concatenated *ITS*-*TUB2*-*RPB2* sequences. (**B**) Phylogenetic tree of Fs64 based on concatenated *ITS*-TEF1α-*RPB2* sequences. The red triangles indicate the positions of El532 and Fs64 in the phylogenetic tree. Green highlights the species that El532 clustered with, i.e., *Epicoccum latusicollum*, while blue highlights the species that Fs64 clustered with, i.e., *Fusarium sacchari*.

Molecular identification based on multi-gene sequence analysis was performed to clarify the taxonomic status of isolates El532 and Fs64 (Figure 4). ITS, TUB2, and RPB2 genes in El532 were amplified, while the ITS, TEF1*α*, and RPB2 genes in Fs64 were amplified. BLAST homology analysis of the obtained sequences in NCBI revealed that the ITS, TUB2, and RPB2 sequences of El532 shared over 99% homology with those of *Epicoccum latusicollum*, and the ITS, TEF1*α*, and RPB2 sequences of Fs64 shared more than 99% homology with *Fusarium sacchari*. Phylogenetic trees were constructed using the maximum likelihood (ML) method based on the concatenated ITS-TUB2-RPB2 sequences for El532 and the concatenated ITS-TEF1*α*-RPB2 sequences for Fs64, together with the corresponding sequences of the closely related species retrieved from GenBank. Notably, phylogenetic analysis revealed that isolate El532 clustered with *E. latusicollum* (Figure 4A), while isolate Fs64 clustered with *F. sacchari* (Figure 4B), confirming their respective close phylogenetic relationships. Isolates El532 and Fs64 were thus definitively identified as *E. latusicollum* and *F. sacchari*, respectively, based on the combination of morphological characteristics, gene sequence homology comparisons, and phylogenetic clustering.

### 3.4. Effects of Carbon and Nitrogen Sources on Pathogen Growth

**Figure 5:**
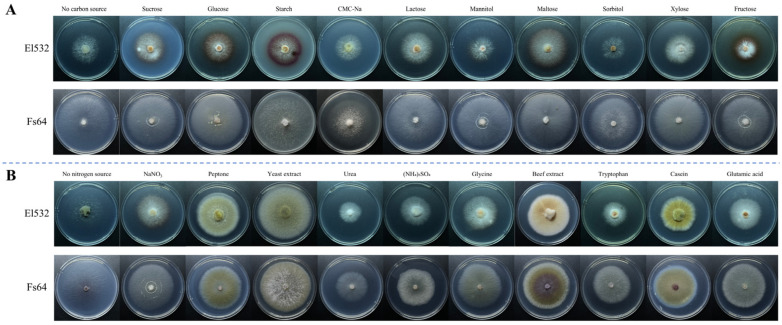
Effects of different carbon and nitrogen sources on colony growth of El532 and Fs64. (**A**) Effects of different carbon sources on colony growth of El532 and Fs64, respectively. (**B**) Effects of different nitrogen sources on colony growth of El532 and Fs64, respectively.

There were significant differences in the mycelial growth of isolates El532 and Fs64 under different carbon sources (Figure 5A, Table 2). For El532, optimal growth occurred on maltose (5.65 ± 0.18 cm), which was significantly higher than on other carbon sources, followed by starch and xylose. Growth on mannitol, sorbitol, and fructose was slow and significantly lower than that on the carbon-free control. In contrast, Fs64 utilized lactose (7.92 ± 0.03 cm), starch (7.90 ± 0.31 cm), and maltose (7.73 ± 0.02 cm) more effectively, with diameters significantly higher than those on other carbon sources. Moreover, there were clear differences in the morphology of colonies of both isolates grown on media with different carbon sources and the carbon-free medium. For instance, El532 had red pigmentation at the colony margins on media containing glucose, starch, and fructose, while Fs64 exhibited more abundant mycelia on media containing starch and CMC-Na.

Nitrogen sources also significantly regulated mycelial growth of both isolates (Figure 5B, Table 2). El532 grew best on yeast extract (7.82 ± 0.23 cm), followed by peptone and beef extract, while growth was poor on tryptophan and urea. Similarly, Fs64 utilized yeast extract most effectively (7.17 ± 0.35 cm), although this was not significantly different from the nitrogen-free control (7.08 ± 0.02 cm). Growth of Fs64 was also significantly inhibited by tryptophan and urea. Notably, compared with the nitrogen-free control, colonies of both isolates grown on all nitrogen-supplemented media exhibited more abundant and denser mycelia, whereas mycelia were sparse and scarce in the nitrogen-free control, indicating that nitrogen sources are essential for mycelial growth and development.

### 3.5. Effects of Temperature and pH on Pathogen Growth

**Figure 6:**
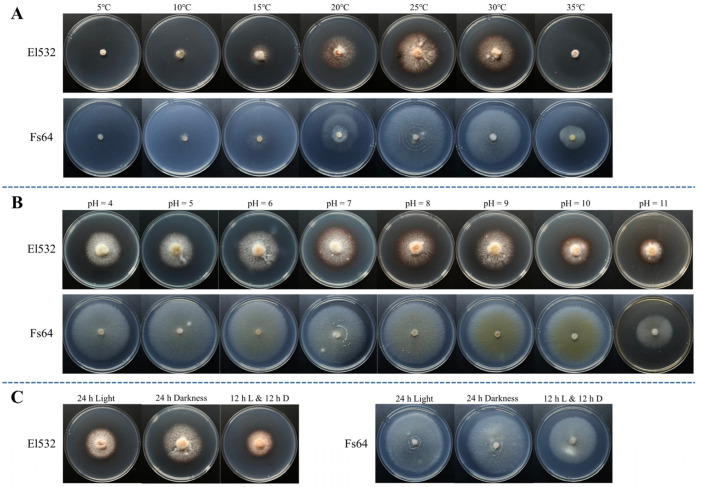
Effects of different temperatures, pH values, and light regimes on colony growth of El532 and Fs64. (**A**) Effects of different temperatures on colony growth of El532 and Fs64, respectively. (**B**) Effects of different pH values on colony growth of El532 and Fs64, respectively. (**C**) Effects of different light regimes on colony growth of El532 and Fs64, respectively.

Colony growth under varying temperatures exhibited significant gradient effects on the mycelial growth of isolates El532 and Fs64 (Figure 6A, Table 3). Notably, the growth of both isolates was inhibited under extremely low and high temperatures. The colony diameter initially increased and then decreased with increasing temperature. Mycelial growth of both isolates was almost arrested at 5 °C but accelerated with increasing temperature between 10 and 25 °C, reaching the maximum colony diameters at 25 °C. The colony expansion rate decreased significantly at 30 °C but remained relatively high, second only to the expansion rate at 25 °C. However, the growth of both isolates was significantly inhibited at 35 °C, especially for El532 (1.05 ± 0.05 cm).

Both isolates grew across the pH range of 4–11 but exhibited significant differences in growth under varying pH conditions (Figure 6B, Table 3). Isolate El532 grew stably at pH 6–9, with the colony diameter significantly higher than that at other pH levels. However, colony growth was significantly inhibited under strongly acidic (pH 4–5) and strongly alkaline (pH 10–11) conditions. In contrast, isolate Fs64 grew well at pH 5–10, with optimal growth at pH 5, reaching a colony diameter of 7.63 ± 0.03 cm. Colony growth was significantly inhibited at pH 4 and pH 11, with the smallest colony diameter recorded at pH 11 (4.52 ± 0.28 cm).

### 3.6. Effects of Light Regime on Pathogen Growth

Different light regimes significantly affected the growth of both isolates (Figure 6C, Table 4). Isolate El532 grew best under continuous darkness, attaining a colony diameter of 5.18 ± 0.20 cm, which was significantly higher than the colony diameter under the other light regimes. El532 exhibited the poorest growth under alternating light/dark conditions, with a colony diameter of only 3.55 ± 0.05 cm. The growth of Fs64 was similar under continuous light and continuous darkness, while alternating light/dark conditions yielded the lowest colony diameter of 6.00 ± 0.15 cm.

### 3.7. Inhibition Effects of Antagonistic Bacteria on Pathogens

**Figure 7:**
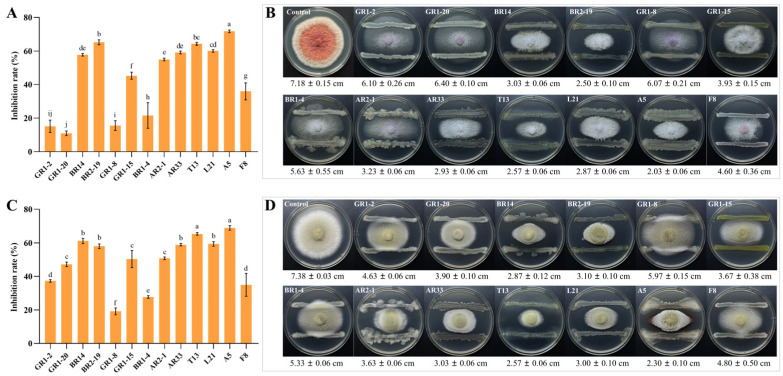
Inhibition effects of antagonistic bacteria against El532 and Fs64, respectively. (**A**) Bar chart of the inhibition rates of different antagonistic bacteria against El532. (**B**) Dual cultures of antagonistic bacteria and El532 on PDA. (**C**) Bar chart of the inhibition rates of different antagonistic bacteria against Fs64. (**D**) Dual cultures of antagonistic bacteria and Fs64 on PDA. The values below each PDA plate indicate the colony diameter. Different lowercase letters indicate significant differences at *p* < 0.05.

Bacteria were isolated from healthy leaves, roots, and rhizosphere soil of sugarcane in the field, followed by screening of antagonistic bacteria using the dual culture method to obtain biocontrol resources against sugarcane leaf spot caused by the two pathogens. A total of 51 bacterial strains were obtained, among which 13 strains exhibited varying inhibitory activity (Figure 7). These 13 strains significantly suppressed the colony expansion of the pathogens compared with the control. Some strains formed clear and stable inhibition zones, resulting in atrophy, deformity, and slowed mycelial growth of the pathogenic fungi. The inhibition rates of the antagonistic strains against El532 ranged from 19.19% to 68.85%, with 6 strains exhibiting inhibition rates higher than 58%. Strain A5 exhibited the highest inhibition rate of 68.85% (Figure 7A,B). In contrast, the inhibition rates against Fs64 ranged from 10.9% to 71.69%, with 4 strains exceeding 60%. Strain A5 exhibited the best inhibitory effect, with an inhibition rate of 71.69%, followed by BR2-19, T13, and L21 (Figure 7C,D). Notably, strains A5, T13, L21, AR33, BR14, and BR2-19 exhibited inhibition rates of over 57% against El532 and Fs64, which underscored their broad-spectrum and stable antagonistic activity. These strains were thus considered potential biocontrol candidates for controlling the causal agents of sugarcane leaf spot disease.

### 3.8. Molecular Identification of Antagonistic Bacteria

Molecular identification based on *16S rRNA* gene sequence analysis was performed to clarify the taxonomic status of the 13 screened antagonistic bacterial strains with inhibitory activity against El532 and Fs64. The 13 strains were classified into four genera: *Bacillus*, *Pseudomonas*, *Enterobacter*, and *Serratia* (Table 5). Notably, *Bacillus* was the dominant genus, comprising 8 strains: *Bacillus velezensis* (BR14), *Bacillus safensis* (GR1-15), *Bacillus pumilus* (AR2-1), *Bacillus subtilis* (BR1-4 and A5), *Bacillus sonorensis* (AR33), *Bacillus altitudinis* (T13), and *Bacillus amyloliquefaciens* (L21). The sequence identity of all *Bacillus* strains with the corresponding reference sequences in GenBank ranged between 99.30% and 100%. Strains GR1-2 and GR1-20 were identified as *Enterobacter asburiae*, with sequence identities of 99.79% and 99.93%, respectively. In contrast, strains GR1-8 and BR2-19 were identified as *Pseudomonas putida* and *Pseudomonas aeruginosa*, respectively, both with 100% sequence identity. Strain F8 had 99.86% sequence identity to *Serratia nematodiphila*.

## 4. Discussion

Sugarcane leaf spot is a prevalent foliar disease that severely impairs sugarcane quality and yield. Despite its economic significance, its management is impeded by the diversity of causal pathogenic fungi and their complex biological characteristics. In this study, *E. latusicollum* (El532) and *F. sacchari* (Fs64) were identified as the causal pathogens of sugarcane leaf spot in Guangxi, China, based on morphological observation, multi-gene sequence analysis, and pathogenicity assays. *E. latusicollum* is known to cause foliar diseases in various crops, including maize (*Zea mays* L.), Chinese ground orchid (*Bletilla striata* (Thunb.) Rchb.f.), and Chinese yam (*Dioscorea polystachya* Turcz.) [14,33,34]. To our knowledge, this is the first report of *E. latusicollum* causing sugarcane leaf spot in China. *F. sacchari* is a member of the *Fusarium fujikuroi* species complex associated with pokkah boeng, *Fusarium* wilt, and red rot of sugarcane in multiple regions [9,35,36,37]. This study confirmed that *F. sacchari* is also a causal pathogen of sugarcane leaf spot, thereby expanding the known disease spectrum of *F. sacchari* as well as its diverse pathogenicity to different sugarcane tissues. Previously, *F. sacchari* has been reported to cause leaf spot in *Dendrobium antennatum* Lindley and *Ananas comosus* (L.) Merr. [38,39]. The identification of these pathogens provides a theoretical basis for epidemiological studies, accurate diagnosis, and green control of sugarcane leaf spot disease.

The occurrence and development of plant diseases are closely associated with environmental conditions. The biological characteristics of pathogens directly determine their environmental adaptability and the regularity of disease occurrence. Analyzing the primary environmental factors affecting their growth is thus crucial for predicting disease epidemics and formulating targeted control strategies [17,40]. Herein, El532 and Fs64 exhibited generally similar temperature response trends, with an optimal growth temperature of 25 °C. Both isolates also grew well at 30 °C. This temperature range corresponds to the typical temperatures during the sugarcane growing season in Guangxi, which potentially contributes to the frequent occurrence of sugarcane leaf spot in the region. This finding is also consistent with the previously reported optimal growth temperature of *Stagonospora tainanensis*, the causal agent of sugarcane leaf blight in Guangxi, China [41]. Moreover, the optimal growth temperature of Fs64 aligns with that of its congeneric pathogens *F. acuminatum* and *F. equiseti* [42,43]. El532 grew well under neutral to weakly alkaline conditions (pH 6–9), which is similar to the pH adaptability reported for *S. tainanensis* (pH 6–8) [41]. In contrast, Fs64 grew well over a broader pH range of 6–10, indicating its strong adaptability to both acidic and alkaline conditions. Pathogen growth was also significantly affected by the light regime. El532 grew best under continuous darkness, while Fs64 grew well under continuous light and continuous darkness. Notably, the alternating light/dark treatment inhibited the growth of both isolates. This phenomenon suggests that light regulation holds potential application value in the control of plant diseases [44].

In addition, both isolates utilized a wide range of carbon and nitrogen sources, reflecting their metabolic versatility. Pathogen virulence is closely associated with the metabolic capabilities of carbon and nitrogen [45]. Herein, maltose was the optimal carbon source for El532, followed by xylose and starch, while Fs64 exhibited the best growth on maltose, starch, and lactose. Yeast extract was the most efficient nitrogen source, promoting significant growth of both isolates. Peptone and beef extract also significantly enhanced the growth of El532. Notably, colonies grown on media supplemented with carbon and nitrogen sources exhibited distinct morphological differences and denser mycelia compared to the carbon-free and nitrogen-free controls, indicating that exogenous carbon and nitrogen sources are essential for the normal growth and development of these pathogens. These nutritional characteristics potentially enhanced their pathogenicity and colonization ability in sugarcane tissues. These findings collectively provide a theoretical reference for understanding niche differentiation, the development of nutrient regulation strategies, and targeted biocontrol approaches.

Biocontrol of sugarcane leaf spot disease using antagonistic microorganisms is a vital alternative to chemical control. In this study, 13 bacterial strains exhibiting antagonistic activity against *E. latusicollum* and *F. sacchari* were isolated from healthy sugarcane tissues and rhizosphere soil. These strains were classified into four genera: *Bacillus*, *Pseudomonas*, *Enterobacter*, and *Serratia*. *Bacillus* species, such as *B. subtilis*, *B. velezensis*, and *B. amyloliquefaciens*, are well known for inhibiting plant pathogens through multiple mechanisms, such as production of antibiotics, extracellular lyase secretion, nutrient competition, and induction of systemic resistance [21,32,46]. Notably, *B. velezensis* has been used to control sugarcane smut and red rot; however, its application against the causal agents of sugarcane leaf spot has not yet been reported [22,25]. Several *Bacillus* strains identified in this study are reported as effective biocontrol agents for pathogens causing plant leaf spot diseases. For instance, *B. subtilis* YLX6 is a highly promising biocontrol agent for controlling the causal agent of blue honeysuckle leaf spot [47], while *B. amyloliquefaciens* TA-1 exhibits significant biocontrol efficacy against the causal agent of peanut early leaf spot [48]. These findings support the biocontrol potential of *Bacillus* strains obtained in this study. Notably, strains A5, T13, L21, AR33, BR14, and BR2-19 exhibited inhibition rates exceeding 57% against both pathogens. *B. subtilis* A5 exhibited the highest inhibition rates (68.85% against El532 and 71.69% against Fs64), making it a promising candidate strain for further development. *Pseudomonas* and *Serratia* species are also recognized as effective biocontrol agents [49,50]. These antagonistic bacteria were isolated from sugarcane roots, leaves, and rhizosphere soil, indicating that various plant-associated microenvironments harbor potential biocontrol agents against sugarcane leaf spot pathogens. The screening of these antagonistic bacteria expands the resource pool of potential biocontrol agents for the causal agents of sugarcane leaf spot disease. However, their biocontrol efficacy against the fungi that cause sugarcane leaf spot will be further evaluated through pot and field experiments in subsequent studies.

## 5. Conclusions

Two pathogenic fungal isolates, El532 and Fs64, identified as *E. latusicollum* and *F. sacchari*, respectively, were isolated from sugarcane leaf spot samples collected in Guangxi, China. This study presents the first report of *E. latusicollum* and *F. sacchari* causing sugarcane leaf spot in China. Subsequent assays to determine the biological characteristics, including temperature, pH, light regimes, and carbon and nitrogen sources of the two pathogens, revealed their environmental adaptability. In addition, 13 antagonistic bacterial strains drawn from four genera were isolated from healthy sugarcane tissues and rhizosphere soil. Among them, *B. subtilis* A5 exhibited the strongest inhibitory activity, with inhibition rates of 68.85% against El532 and 71.69% against Fs64. The findings of this study provide a foundation for sugarcane leaf spot management. Future studies should focus on developing control strategies for sugarcane leaf spot disease based on the clarified biological characteristics of the pathogens and the screened antagonistic bacteria.

## Figures and Tables

**Table 1:** Primer sequences and their annealing temperatures.

Gene	Primers	Tm	Sequences (5′-3′)
*ITS* [28]	ITS1	56 °C	TCCGTAGGTGAACCTGCGG
	ITS4		TCCTCCGCTTATTGATATGC
*TUB2* [29]	T1	56 °C	AACATGCGTGAGATTGTAAGT
	T2		TAGTGACCCTTGGCCCAGTTG
*RPB2* [30]	5f2	57 °C	GGGGWGAYCAGAAGAAGGC
	7cR		CCCATRGCTTGYTTRCCCAT
*TEF1α* [31]	1H	51 °C	ATGGGTAAGGAGGACAAGAC
	2T		GGAAGTACCAGTGATCATGTT
*16S rRNA* [32]	27F	55 °C	AGAGTTGATCCTGGCTCAG
	1492R		GTTACCTTGTTACGACTT

**Table 2:** Colony diameters of El532 and Fs64 under different carbon and nitrogen sources. Different lowercase letters indicate significant differences at *p* < 0.05.

Carbon Source Utilization Assay			Nitrogen Source Utilization Assay		
Treatment	El532 (cm)	Fs64 (cm)	Treatment	El532 (cm)	Fs64 (cm)
No carbon	4.37 ± 0.10 d	7.12 ± 0.13 bc	No nitrogen	5.32 ± 0.23 c	7.08 ± 0.20 a
Sucrose	4.72 ± 0.13 c	7.08 ± 0.18 bc	NaNO_3_	5.08 ± 0.03 c	6.90 ± 0.05 ab
Glucose	4.32 ± 0.08 d	7.28 ± 0.24 bc	Peptone	6.30 ± 0.35 b	6.45 ± 0.05 d
Starch	5.12 ± 0.10 b	7.90 ± 0.31 a	Yeast extract	7.82 ± 0.23 a	7.17 ± 0.35 a
CMC-Na	4.18 ± 0.03 de	7.02 ± 0.08 c	Urea	3.07 ± 0.20 f	5.05 ± 0.43 f
Lactose	4.80 ± 0.22 c	7.92 ± 0.03 a	(NH_4_)_2_SO_4_	4.48 ± 0.10 d	5.40 ± 0.50 ef
Mannitol	4.02 ± 0.08 e	7.38 ± 0.28 b	Glycine	5.07 ± 0.06 c	6.68 ± 0.10 bcd
Maltose	5.65 ± 0.18 a	7.73 ± 0.20 a	Beef extract	6.50 ± 0.09 b	6.83 ± 0.08 abc
Xylose	5.08 ± 0.08 b	7.12 ± 0.23 bc	Tryptophan	3.67 ± 0.08 e	5.67 ± 0.10 e
Sorbitol	3.72 ± 0.18 f	7.20 ± 0.05 bc	Casein	5.25 ± 0.09 c	6.55 ± 0.13 bcd
Fructose	3.82 ± 0.03 f	7.12 ± 0.13 bc	Glutamic acid	4.57 ± 0.33 d	6.50 ± 0.13 cd

**Table 3:** Colony diameters of El532 and Fs64 under different temperatures and pH values. Different lowercase letters indicate significant differences at *p* < 0.05.

Temperature Assay			pH Assay		
Temperature (°C)	El532 (cm)	Fs64 (cm)	pH	El532 (cm)	Fs64 (cm)
5.0	0.72 ± 0.03 f	0.72 ± 0.03 g	4.0	4.43 ± 0.06 b	6.82 ± 0.03 c
10.0	1.93 ± 0.10 d	1.57 ± 0.10 f	5.0	4.65 ± 0.05 b	7.63 ± 0.03 a
15.0	3.15 ± 0.09 c	2.77 ± 0.06 e	6.0	4.92 ± 0.08 a	7.28 ± 0.31 b
20.0	4.77 ± 0.18 b	4.57 ± 0.20 c	7.0	5.00 ± 0.20 a	7.25 ± 0.09 b
25.0	5.30 ± 0.09 a	7.55 ± 0.13 a	8.0	5.03 ± 0.06 a	7.43 ± 0.15 ab
30.0	4.88 ± 0.03 b	6.83 ± 0.10 b	9.0	4.95 ± 0.05 a	7.45 ± 0.09 ab
35.0	1.05 ± 0.05 e	3.18 ± 0.16 d	10.0	3.95 ± 0.18 c	7.28 ± 0.14 b
			11.0	3.13 ± 0.32 d	4.52 ± 0.28 d

**Table 4:** Colony diameters of El532 and Fs64 under light regimes. Different lowercase letters indicate significant differences at *p* < 0.05.

Treatment	El532 (cm)	Fs64 (cm)
24 h Light	4.10 ± 0.05 b	7.57 ± 0.29 a
24 h Darkness	3.55 ± 0.05 c	7.28 ± 0.08 a
12 h L and 12 h D	5.18 ± 0.20 a	6.00 ± 0.15 b

**Table 5:** Source and Molecular Identity of Antagonistic Bacteria.

Antagonistic Bacteria	Source	Identification	Percent Identity
GR1-2	Root	*Enterobacter asburiae*	99.79%
GR1-20	Root	*Enterobacter asburiae*	99.93%
BR14	Root	*Bacillus velezensis*	100%
BR2-19	Root	*Pseudomonas aeruginosa*	100%
GR1-8	Root	*Pseudomonas putida*	100%
GR1-15	Root	*Bacillus safensis*	100%
AR2-1	Leaf	*Bacillus pumilus*	99.85%
BR1-4	Leaf	*Bacillus subtilis*	100%
AR33	Leaf	*Bacillus sonorensis*	99.30%
T13	Soil	*Bacillus altitudinis*	100%
F8	Soil	*Serratia nematodiphila*	99.86%
A5	Soil	*Bacillus subtilis*	99.86%
L21	Soil	*Bacillus amyloliquefaciens*	99.44%

## Data Availability

The original contributions presented in this study are included in the article. Further inquiries can be directed to the corresponding authors.
